# Refractory type 1 plastic bronchitis in a child; case report

**DOI:** 10.1186/s12887-024-04893-x

**Published:** 2024-07-10

**Authors:** Rehab Elmeazawy, Ahmed Elniny

**Affiliations:** Department of Pediatrics, Faculty of Medicine, Tanta University, Tanta, Egypt

**Keywords:** Plastic, Bronchitis, Casts, Child, Refractory

## Abstract

**Background:**

Plastic bronchitis (PB) is a rare pediatric pulmonary condition characterized by the production of branching bronchial casts that cause partial or total obstruction of the bronchial lumen.

**Case presentation:**

We describe a 13-year-old boy with a history of bronchial asthma and left lower lobectomy, with persistent cough and left-sided chest pain when he went to the emergency room. Chest radiography showed complete left lung opacity denoting total left lung collapse, and flexible bronchoscopy revealed cohesive casts totally occluding the left bronchus, with frequent recurrence that finally ended with left pneumonectomy.

**Conclusion:**

Plastic bronchitis is a rare, fatal disease in children that requires a high index of suspicion for both diagnosis and treatment. Although bronchoscopic removal of the bronchial casts together with the medical treatment are the main lines of treatment, cases with recurrent formation of casts are at high risk for surgical intervention in the form of either lobectomy or pneumonectomy.

## Background

Plastic bronchitis is a rare disease characterized by the formation of branching cohesive casts that occlude the bronchial tree, either partially or completely. The typical clinical presentations of PB are symptoms of lower respiratory tract infections, including fever and cough, during which patients may expectorate the casts, dyspnea, wheezing, and chest pain. Sometimes, patients may exhibit a flag-flapping sound (bruit de drapeau) due to the movement of the casts during inspiration and expiration [1].

Plastic bronchitis is classified into two types according to the constituents of the formed casts: Type 1 PB is characterized by the formation of highly cellular inflammatory casts, mainly eosinophilic cells, and is usually associated with diseases such as asthma, cystic fibrosis, non-cystic fibrosis bronchiectasis, sickle cell disease, and smoke inhalation, while type 2 PB is composed of acellular materials composed of fibrin and mucin, which is usually linked to post-surgical correction of congenital heart diseases [2].

Eosinophilic plastic bronchitis is a distinct subtype of plastic bronchitis, predominantly found in the pediatric population, with limited representation in current literature. Some patients might possess a medical background involving asthma or atopy, although a significant proportion may not exhibit such history. The clinical presentation may vary from mild symptoms up to complete lung collapse mimicking the symptoms of foreign body aspiration, thus necessitating a high index of suspicion for the diagnosis of PB in order to effectively manage it and prevent future recurrences.

We describe the case of a male child with a history of acute bronchial asthma treated with optimal asthma medications for biological therapy and complicated by left lower lung bronchiectasis that required lobectomy at the age of 10 years who presented at the age of 13 years with clinical manifestations compatible with PB. In addition to the clinical, laboratory, radiological, and histopathological results, the treatment, and outcome are identified in this case.

## Case presentation

We describe the case of a 13-year-old boy who presented with persistent cough and left-sided chest pain when he went to the emergency room. Since the age of five months, bronchopneumonia episodes have often occurred, requiring frequent hospital admissions. Acute bronchial asthma was identified in the child, and treatment with bronchodilators and inhaled steroids resulted in only partial improvement.

**Fig. 1:**
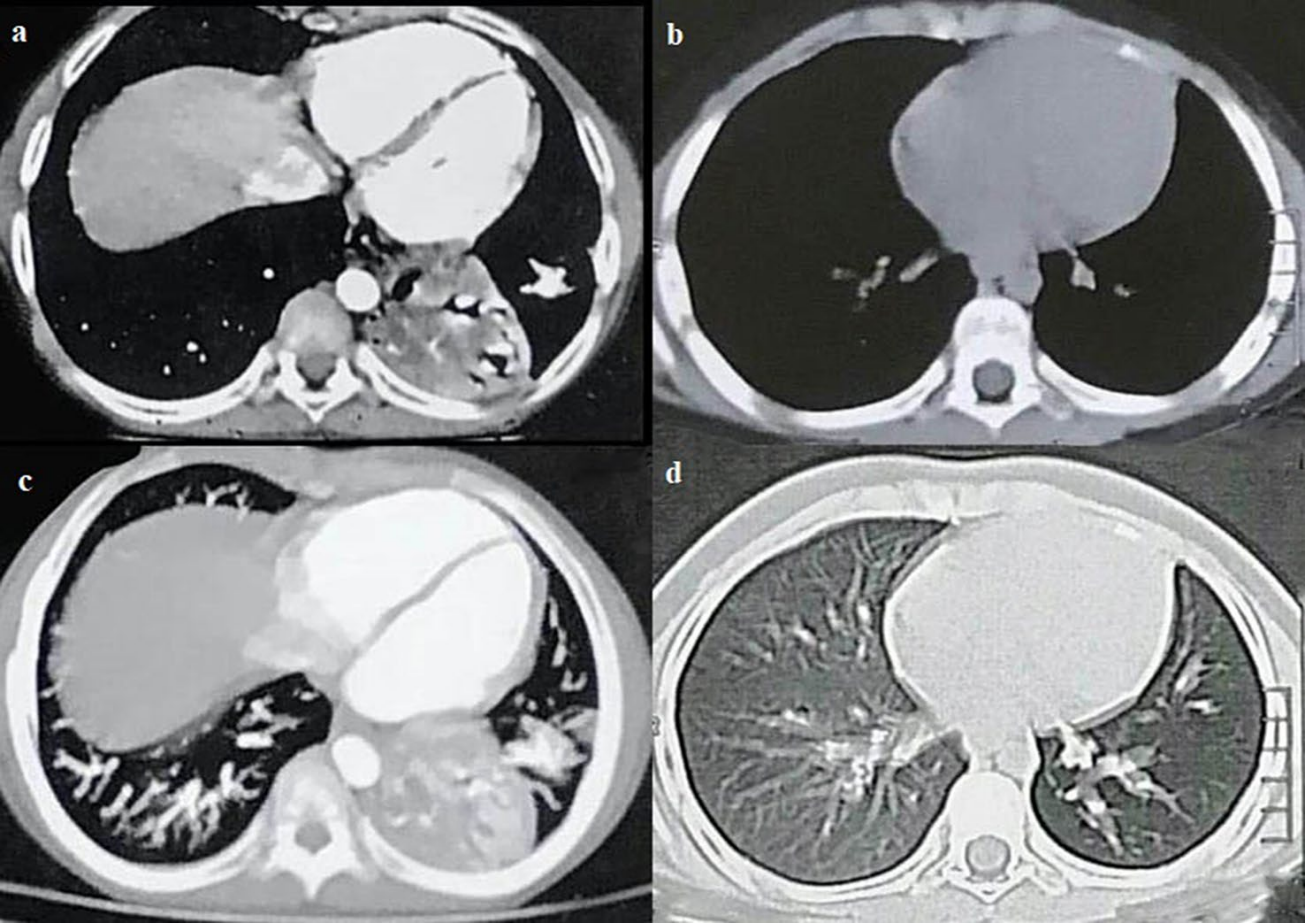
(**a**) and (**b**). mediastinal and lung window of CT chest showing left lower sequestrated lobe, (**d**) and (**e**). mediastinal and lung window of CT chest 6 months after left lower lobectomy

When he was 10 years old, he developed high-grade fever, persistent cough, and shortness of breath that necessitated the use of antibiotics in the hospital. A left lower sequestrated lobe was identified on the CT scan of the chest, prompting the intervention of a cardiothoracic surgeon who proceeded with a left lower lobectomy (Fig. 1). This surgical procedure was deemed necessary due to the patient’s recurrent fever, chest pain, and the requirement for frequent administration of intravenous antibiotics. Unfortunately, the histopathology from his lobectomy was unavailable.

After a year of clinical improvement, the child developed a persistent productive cough and recurrent left-sided chest pain that did not improve with asthma treatment.

Physical examination showed a fever 38^0^c, respiratory rate of 25 breaths/min, heart rate of 110 beats/min, and blood pressure of 110/70. Oxygen saturation was 95% in room air. The patients’ heights and weights were within the median range. He had a slight intellectual disability. An examination of the left side of the chest revealed no air entry.

**Fig. 2:**
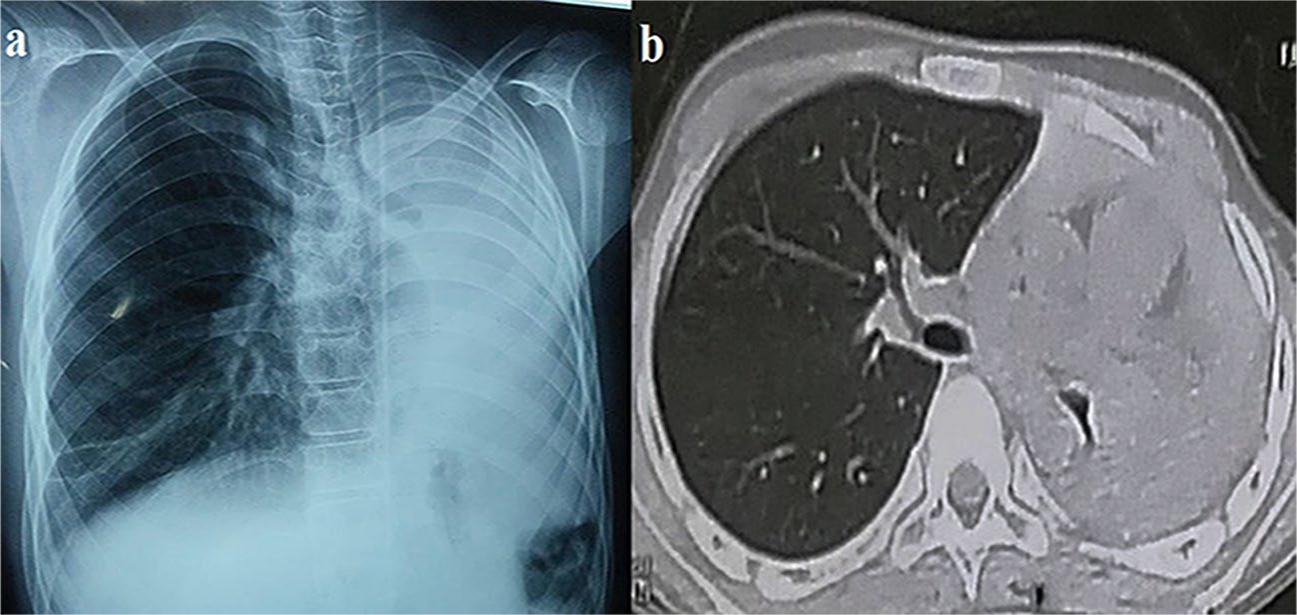
(**a**) Chest radiograph, and (**b**) CT chest showing left hemithorax opacification

The left hemithorax was completely opaque on chest radiography, the mediastinum was shifted to the same side, and hyperinflation of the right lung suggested that the left lung had collapsed. The CT scan confirmed the chest X-ray’s findings (Fig. 2). CT of the maxillary sinuses showed mild sinusitis. Echocardiography revealed a heart with a normal architecture and function.

Upon admission, laboratory results revealed normal complete blood count (CBC) and serum biochemistry, C-reactive protein (CRP) level of 10 mg/L (up to 6 mg/L), normal erythrocyte sedimentation rate (ESR), and negative sputum culture.

**Fig. 3:**
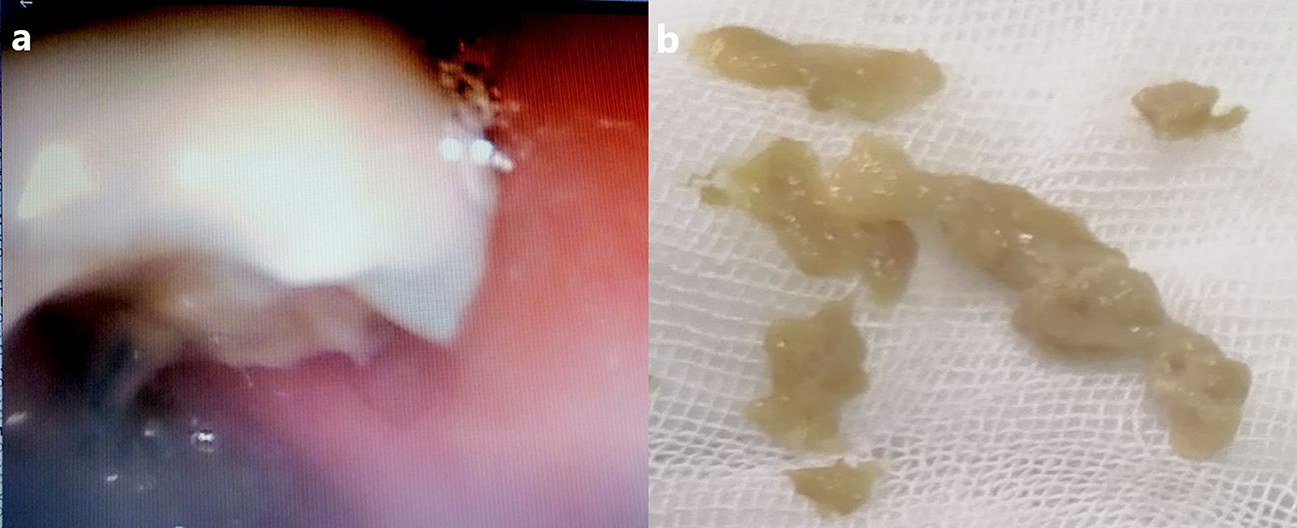
(**a**) Bronchoscopic image of whitish fleshy cast occluding left bronchus, (**b**) Macroscopic appearance of the bronchial casts

**Fig. 4:**
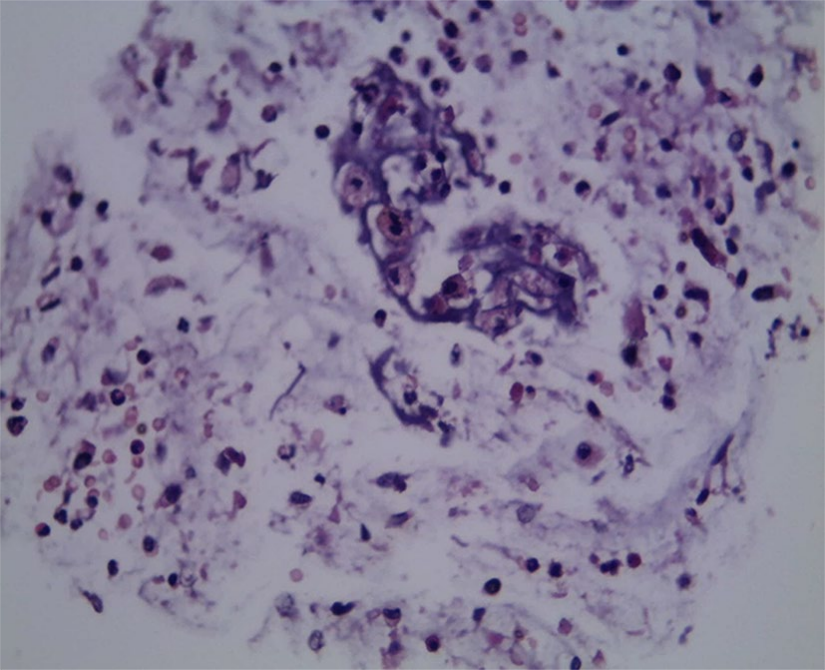
The microscopic appearance of the cast is a mix of large numbers of eosinophils and a few other inflammatory cells in a mucinous background. Hematoxylin-eosin stain, original magnification × 200

On the sixth day after admission, flexible fiber-optic bronchoscopy was performed to remove several whitish-fleshy tissues that appeared to be foreign bodies from the left main bronchus (Fig. 3). Mild left bronchomalacia and significant bronchial tree inflammation beneath the bronchial cast were observed. Bronchoalveolar lavage (BAL) cell count showed elevated neutrophils (55%), lymphocytes (35%), eosinophils (10%), and no bacterial growth on BAL culture. Histopathology of the casts revealed inspissated secretions with significant eosinophilic infiltration consistent with type 1 inflammatory casts (Fig. 4).

**Fig. 5:**
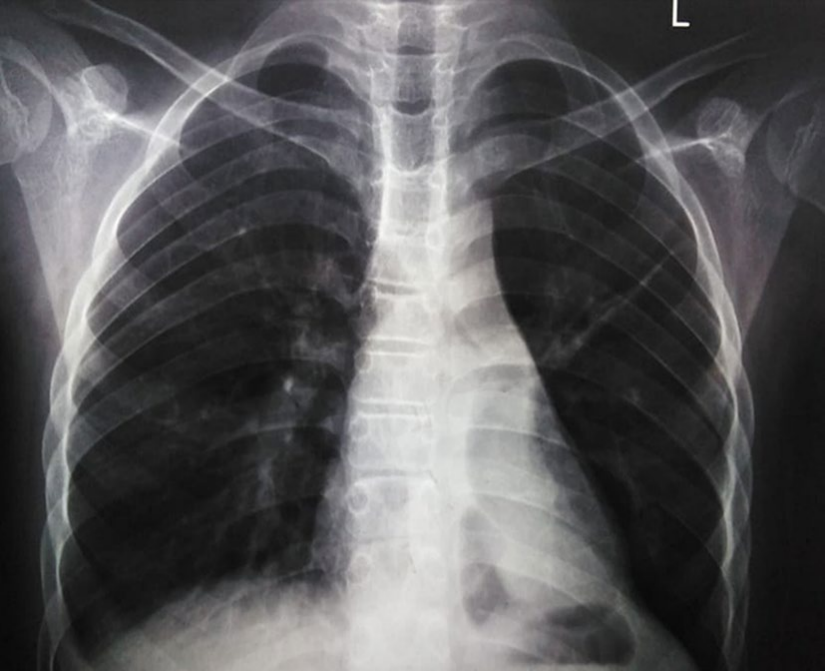
Chest X-ray showing a normal left lung at 24 h. after a flexible bronchoscopy

After using a bronchoscope for 24 h, we performed a chest X-ray, which revealed satisfactory left lung inflation and a central mediastinum (Fig. 5). Immunoglobulin E was measured and found to be elevated at 177 IU/ml (< 90); Aspergillus fumigatus IgE results were negative, and tuberculosis was ruled out as a potential cause of cast development.

Laboratory analyses were conducted in order to investigate the underlying etiology of the eosinophilic cast such as sweat chloride test and genetic testing for cystic fibrosis which were negative. Additionally, hemoglobin electrophoresis and High-performance liquid chromatography (HPLC) were performed to rule out sickle cell diseases.

Three weeks later, the patient was discharged and received controller medication for allergic rhinitis and asthma in the form of intranasal steroids, long-acting B2 agonists (LABA), mucolytics, and inhaled steroids.

Although we performed both flexible and rigid bronchoscopy to remove the occluding bronchial casts using topical N-acetylcysteine, heparin, and recombinant human tissue-type plasminogen activator (tPA), the child returned five times with a completely collapsed left lung. None of these medications were able to stop recurrence.

We added omalizumab to the asthma controller therapy, but the patient still had the same clinical and radiological findings, since his compliance with proper chest physiotherapy was very poor due to intellectual disabilities.

**Fig. 6:**
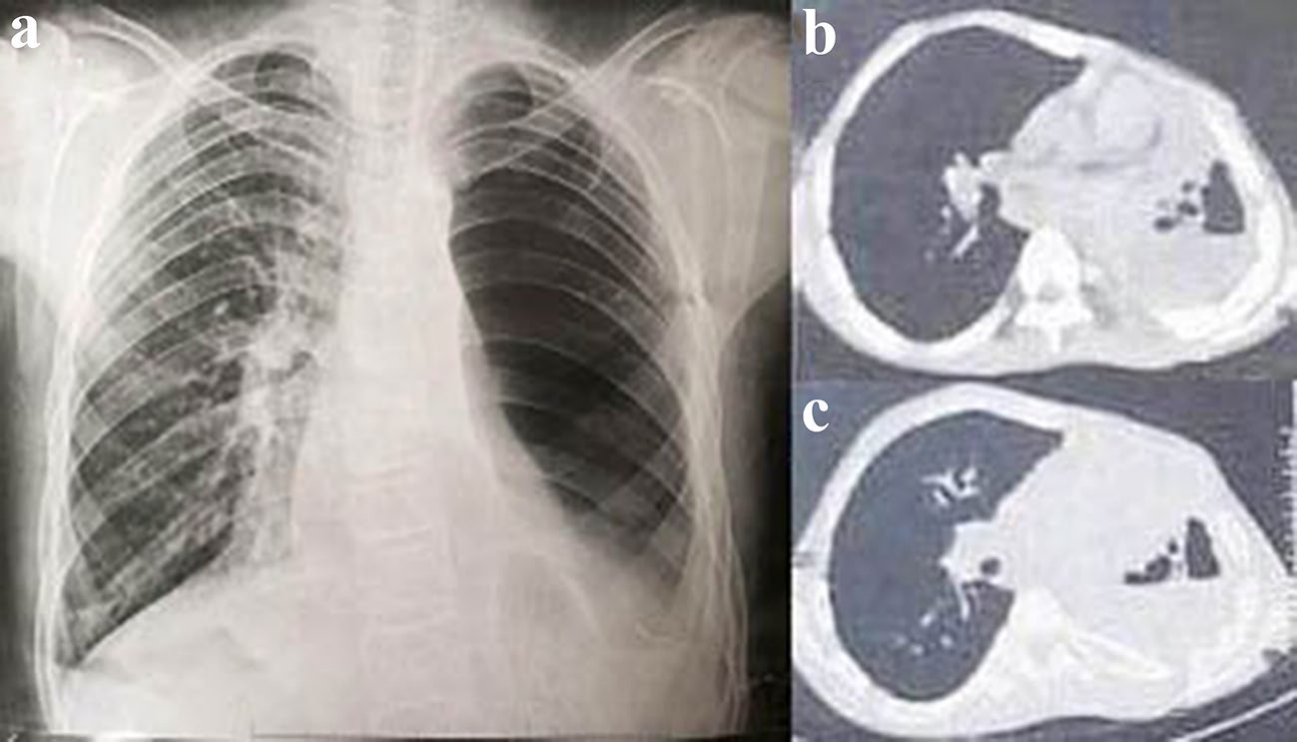
(**a**) Chest radiography shows gas filling the left hemithorax after pneumonectomy just after the surgery, (**b**) and (**c**) mediastinal and lung window of CT chest shows partial gas filling the left hemithorax one year after the surgery

Because of the refractory nature of the casts despite the use of inhaled and topical therapies (inhaled hypertonic saline, N-acetylcysteine, heparin, and tPA) and optimization of asthma control with omalizumab, cardiothoracic surgeons recommended left pneumonectomy to alleviate the patient’s symptoms (Fig. 6), and his follow-up indicated greater clinical improvement without hospitalization since the procedure.

Histopathological examination of the surgically removed lung tissue revealed massive infiltration of the interstitial and intra-alveolar tissue by inflammatory cells mainly neutrophils, eosinophils, lymphocytes up to formation of lymphoid follicles, macrophages together with the presence of intra-alveolar macrophage, and areas of fibrosis. The interalveolar septa are thickened with congestion of alveolar capillaries. No malignancy was detected in the specimen.

During his regular medical check-ups, spirometry was performed, indicating a mixed pattern (FEV1 42%, FVC 35%, and FEV1/FVC 82%), which exhibited positive response to bronchodilator reversibility testing (FEV1 45%, FVC 40%, and FEV1/FVC 112%). However, it also showed a reduction in comparison to pre-pneumonectomy levels (FEV1 59%, FVC 52%, and FV1/FVC 113%).

Our case illustrates that the current treatment for plastic bronchitis is still insufficient, and surgical intervention may be necessary for patients with recurring plastic bronchitis who do not respond to conventional treatment.

## Discussion and conclusions

We report a case of complete left bronchial obstruction caused by PB in a child with a medical history of bronchial asthma. Bronchial casts are categorized as type I when composed of inflammatory cells and accompanying disorders, such as asthma and pneumonia, and type II when acellular and related to congenital heart abnormalities following operations, such as Fontan [3].

Plastic bronchitis and cast formation have various causes. This comprises lymphatic anomalies, infections, hematological diseases, atopic diseases, cancer, and other conditions. Casts associated with congenital heart disease may be caused by improper lymphatic drainage. The origin of the cast in individuals with asthma is believed to be chronic inflammation and cellular infiltration with neutrophils and eosinophils, which is consistent with our case [4].

The main objectives of plastic bronchitis treatment are long-term avoidance of cast formation and acute removal of casts using flexible or rigid bronchoscopy. N-acetylcysteine, hypertonic saline, and dornase alpha are inhaled molecules that have been utilized to aid in cast disruption [5].

The instillation of N-acetylcysteine during flexible bronchoscopy and via nebulization before and after the procedure proved ineffective in our patient for preventing the recurrence of lung collapse. Kumar et al., in contrast, demonstrated the efficacy of nebulized N-acetylcysteine in averting the reappearance of plastic bronchitis caused by asthma in their two documented cases [6]. The disparity in outcomes could possibly be linked to the age of the patients and the administration of asthma medications prior to the development of complications as observed in our patient.

Tissue plasminogen activator (tPA) and urokinase inhalation may be an alternative for the lysis of casts made of fibrin tissue that are linked to congenital heart problems; however, they may not be a helpful intervention when asthma or a related cause is present [7]. Gibb et al. discovered that the application of tPA through direct instillation into the airways during bronchoscopy and via a nebulizer demonstrated efficacy and safety as a treatment for plastic bronchitis and effectively averting the development of complications associated with significant airway blockage. Nonetheless, the utilization of tPA in our patient failed to yield encouraging outcomes [8].

Aerosolized heparin has also been used successfully in cast lysis; however, it is unclear whether the effects of heparin are related to its anti-inflammatory characteristics or its effects on thrombolysis via antithrombin III. Furthermore, heparin is less expensive than tissue plasminogen activators and causes less airway irritation [9]. Macrolide antibiotics have also been used in the treatment of plastic bronchitis because of their anti-inflammatory action on the airways [10].

In pediatric patients with type 1 PB, both inhaled and systemic corticosteroids have been shown to be beneficial in lowering inflammatory cast formation and treating the symptoms of plastic bronchitis. Long-term use of low-dose corticosteroids and macrolides has shown a good anti-inflammatory effect in minimizing cast development, particularly in type 1 plastic bronchitis [11].

In addition to different medication therapies, flexible or rigid bronchoscopic removal of the casts has emerged as the gold standard for treating plastic bronchitis because it can both identify the underlying cause and alleviate symptoms [12].

Treatment of the underlying disease is effective in preventing the recurrence of cast formation. Lymphatic embolization, thoracic duct ligation, and thoracic duct stent grafting may be effective in type 2 PB, whereas in rare cases of type 1 PB not responding to optimal medical treatment like in our case lobectomy may be utilized [13].

To the best of our knowledge, no clinical studies have shown that a single treatment is consistently successful regardless of the type of plastic bronchitis.

## Data Availability

All data generated or analyzed during this study are included in this published article.
